# Nucleobase morpholino β amino acids as molecular chimeras for the preparation of photoluminescent materials from ribonucleosides

**DOI:** 10.1038/s41598-020-76297-7

**Published:** 2020-11-09

**Authors:** Raffaella Bucci, Alberto Bossi, Emanuela Erba, Francesco Vaghi, Abhijit Saha, Sivan Yuran, Daniela Maggioni, Maria Luisa Gelmi, Meital Reches, Sara Pellegrino

**Affiliations:** 1 DISFARM-Dipartimento Di Scienze Farmaceutiche, Sezione Chimica Generale E Organica “A. Marchesini”, Università Degli Studi Di Milano, Via Venezian 21, 20133 Milan, Italy; 2 Istituto Di Scienze E Tecnologie Chimiche “G. Natta” del Consiglio Nazionale Delle Ricerche (CNR-SCITEC), via Fantoli 16/15, 20138 Milan, Italy; 3 SmartMatLab Center, via C. Golgi 19, 20133 Milan, Italy; 4 Institute of Chemistry and the Center for Nanoscience and Nanotechnology, The Hebrew University of Jerusalem, Institute of Chemistry, Jerusalem, Israel; 5 Dipartimento Di Chimica, Università Degli Studi Di Milano, Via Golgi 19, 20133 Milan, Italy

**Keywords:** Materials chemistry, Organic chemistry, Supramolecular chemistry

## Abstract

Bioinspired smart materials represent a tremendously growing research field and the obtainment of new building blocks is at the molecular basis of this technology progress. In this work, colloidal materials have been prepared in few steps starting from ribonucleosides. Nucleobase morpholino β-amino acids are the chimera key intermediates allowing Phe–Phe dipeptides’ functionalization with adenine and thymine. The obtained compounds self-aggregate showing enhanced photoluminescent features, such as deep blue fluorescence and phosphorescence emissions.

## Introduction

The development of humankind society has often been measured by the types of material that were used during the ages, from Stone, Iron, Bronze to Steel and, in the second part of the twentieth century, Silicon. In the last two decades, a new evolution of man-made materials has been started, this time powered by nanotechnology. Nanomaterials are thus expected having a strong impact on societal change owing to their wide applications ranging from clean energy to biomedicine^1–3^. This influence on society is paving the basis to the Nanomaterials Age. Consequently, the development of new, biocompatible nanomaterials characterized by enhanced properties and features is a growing, stimulating research field.

**Scheme 1:**
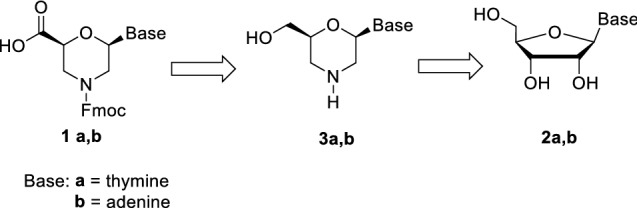
Retrosynthetic pathway to nucleobase morpholino β-amino acids.

Natural biomacromolecules, as proteins and nucleic acids, can create a wide range of nano-assemblies. Amino acids and nucleotides are at the molecular level of this high complexity and, in the recent years, many studies have been focusing on the development of bio-inspired building blocks that self-assemble into preferred architectures^4–10^. One successful example is the dipeptide diphenylalanine can build up different morphologies depending on the environment conditions and functionalization^11,12^. The driving force of diphenylalanine self-assembly is the π–π stacking between the aryl groups that are then enforced by hydrogen bonds stabilizing the final architectures^13–17^. More in general, amino acid and peptide-based supramolecular structures are normally formed through H-bonding, van der Waals force and π–π interactions. These peptide systems are very stable and can withstand high temperature and exposure to chemicals^15,16^. In addition, they possess high mechanical stability^17^. Nucleotide-based architectures are formed due to Watson–Crick interactions allowing specific molecular recognition and base-pairing complementarity. The combination of peptides and nucleic acids could be thus very useful for the design of novel self-organized materials with enhanced properties and features. Nevertheless, nucleopeptides^18^, i.e. peptides containing both nucleobases and amino acids, are still underexploited in the development of functional materials. Few examples are reported in the literature, such as environment polarity driven self-assembly of helical Aib nucleofoldamers^19^ and diphenylalanine nucleopeptides hydrogels^20^. In both examples, the nucleobases are linked to the C- and/or N-terminus of the peptides. A different strategy could be the synthesis of peptides containing nucleo amino acids, i.e. amino acids bearing a nucleobase on the side chain. During the years, several linear α^21,22^ and β^11,12^ nucleo amino acids^23^ have been developed and used for the preparation of tubular or sheet-like aggregates. In this work, we present a new class of β amino acids **1** containing a morpholino ring and a nucleobase. These nucleo amino acids were prepared in enantiopure form from ribonucleosides **2**, through nucleo morpholino alcohols **3**, as the key intermediates (Scheme 1).

Nucleo amino acids **1** were then used for the preparation of ultra-short peptides containing the dipeptide Phe–Phe that showed self-aggregation propensity in water and polar solvents, leading to the obtainment of sub-micrometric spherical morphologies. Finally, given the formally deep-UV active features of the Phe–Phe residue^24,25^, steady state and time resolved photoluminescence (PL) studies have been performed on the synthesized ultra-short peptides revealing their deep blue fluorescence and an unprecedent phosphorescence.

**Scheme 2:**
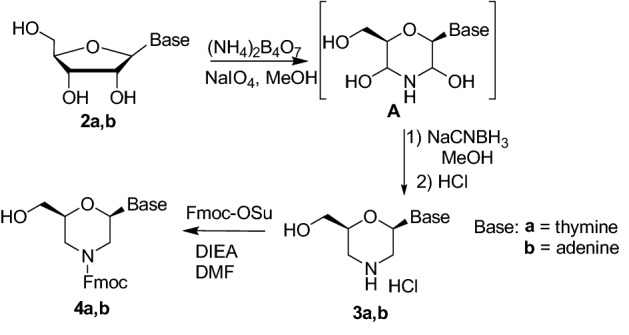
Synthesis of morpholino alcohols **4.**

The synthesis of alcohol intermediates **3** was reported in 1993 by Summerton in a patent^26^ and consisted in a “one-pot” oxidative ring-opening and reductive amination of the ribose sugar leading to the obtainment of the morpholino ring (Scheme 2). Several modifications of this procedure have been reported in the last years^27–30^. Here, we started from unprotected ribonucleosides, using the Summerton’s procedure with different work up conditions. Briefly, the sugar ring is converted into the corresponding morpholino through a “one-pot” oxidation/amination reaction using NaIO_4_ (1.1 eq) and (NH_4_)_2_B_4_O_7_ (1.15 eq) in MeOH. The crude intermediates **A** were directly treated with NaCNBH_3_ (1.7 eq) in MeOH affording compounds **3**. The reaction was quenched with HCl and the products **3a** and **3b** were isolated as hydrochloride salts by crystallization from polar solvents (**3a**: MeOH, 60%; **3b**: ACN, 70%).

**Scheme 3:**
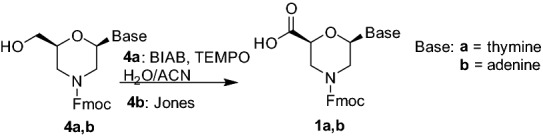
Synthesis of nucleobase morpholino β-amino acids **1**.

We then investigated the oxidation reaction of the hydroxyl group affording to the β-amino acid function. Firstly, the protection of the endocyclic nitrogen was required to avoid side reactions on the free amine. The Fmoc protecting group was selected, in view of using *N*-protected compounds **1** for the preparation of nucleopeptide materials. Fmoc group is indeed known to favor self-assembly through π–π stacking^31^. The reaction was carried out directly on the crude **3a,b** obtained in the previous reaction (Scheme 2) simply adding FmocOSu (1 eq) and DIEA (1–3 eq) in DMF. The target compounds **4a** and **4b** were thus obtained in 85% and 55% yield, respectively. Several oxidants were then tested on the compounds **4a** and **4b** (Table S1 in Supplementary Information). For compound **4a**, having thymine as nucleobase, the combination BIAB/TEMPO^32^ was found the most effective, affording compound **1a** with 90% yield (Scheme 3). On the other hand, in the same conditions, compounds **4b** scarcely reacted and the Fmoc deprotection occurred by prolonging reaction times. Acid **1b** (70%) was thus obtained by Jones oxidation with CrO_3_ (Scheme 3).

**Scheme 4:**
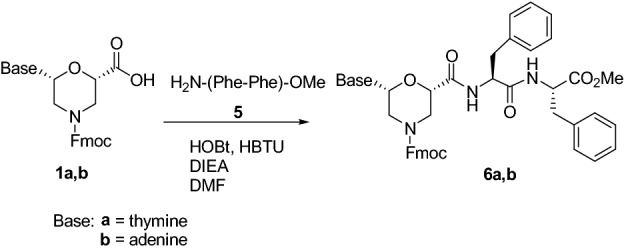
Synthesis of tripeptides **6a,b**.

*N*-Fmoc protected thymine and adenine nucleo amino acids **1a-b** were then used for the preparation of ultra-short nucelopeptides containing diphenylalanine. The coupling with the dipeptide H_2_N–Phe–Phe–OMe **5** was performed using HOBt/HBTU (1.1 eq each), as condensing agents, and DIEA (4 eq), as the base, leading to compounds **6a,b** in 80% yield (Scheme 4).

**Figure 1:**
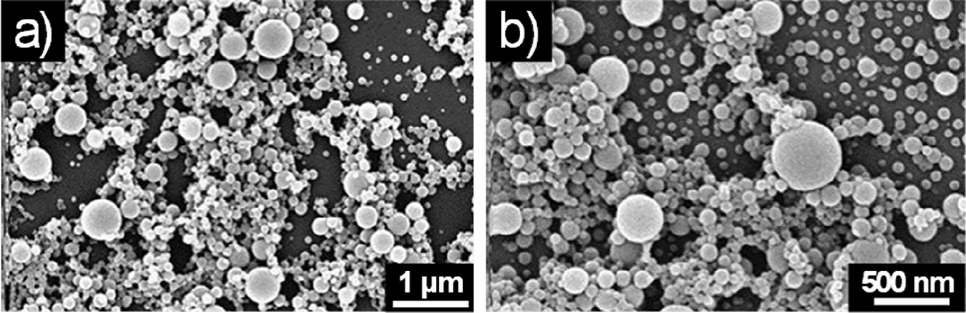
SEM micrographs of the self-assembled structures formed by **6a** (**a**) and **6b** (**b**) in pure water.

The self-assembly behavior of both compounds **6a,b** was studied using the solvent displacement method. A solution of compound **6a,b** in hexafluoroisopropanol (HFIP, 100 mg/mL) was diluted with different solvents (distilled water, EtOH, 50% EtOH, MeOH, isopropanol, chloroform) to a final concentration of 2 mg/mL. The formation of sub-micrometric aggregates was observed by drop-casting on silicon wafers for recording SEM after 30 min and after 24 h (see Supplementary information). In water, both compounds **6a** and **6b** exhibited spherical aggregates (Fig. 1) whose size ranged from a few tens of nanometers to less than a micron. In chloroform, isopropanol and HFIP no aggregates were observed.

**Figure 2:**
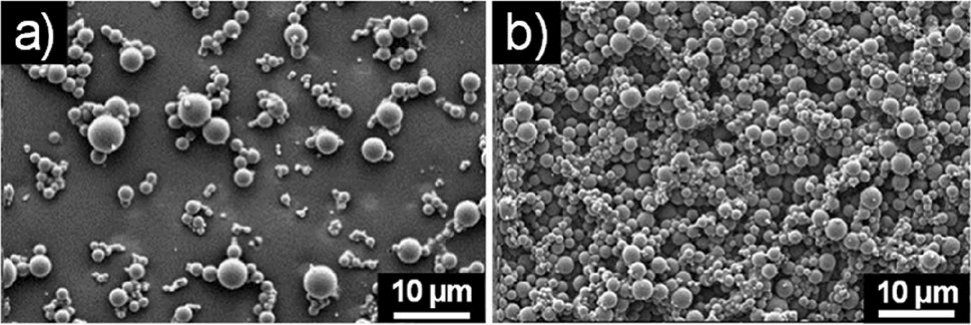
SEM micrographs of the self-assembled structures formed by **6b** (**a**) in EtOH and (**b**) MeOH.

In EtOH and MeOH, **6a** and **6b** had a different behavior. Compound **6a** exhibited indeed spherical macro-aggregates in 50% EtOH (Fig. S1 in Supplementary information), while **6b** self-aggregates in ordered spherical structures both in EtOH and MeOH (Fig. 2).

Considering the promising results obtained in HFIP/ H_2_O, we performed DLS analysis on **6a** and **6b** at different HFIP/H_2_O ratios (see Supplementary information). Our results suggested that the quantity of water is fundamental for determining the formation of aggregates, their size and and their distribution. At the lowest water content (HFIP/H_2_O, 70:30) the formation of nanocolloidal clusters of 5–6 nm was observed together with larger agglomerates. An enhancement in the size of the aggregates was obtained increasing the water amount, although the distribution was not optimal. When the HFIP content was only the 2% the **6a** and **6b** suspensions showed only one sharp peak centered at ca. 200 nm, indicating the formation of a monodispersed colloidal suspension (see Table 1 and Supplementary information). In the case of **6a**, an increase of the size of the agglomerates was observed by measurements repeated after 24 h and 48 h. A negative ζ-potential was detected for both **6a** and **6b**, suggesting a protonated state of the nucleobases with a consequent formation of a tight ion pair due to the presence of acidic HFIP.

**Table 1:** Hydrodynamic diameter by DLS and ζ-potential of **6a** and **6b** aggregates.

H_2_O/HFIP	**6a**	**6b**
98:2 (t=0)	190 ± 60 nm	190 ± 91 nm
98:2 (t=24h)	342 ± 120 nm	220 ± 100 nm
98:2 (t=48h)	342 ± 150 nm	220 ± 100 nm
ζ-potential	− 47 mV	− 16 mV

**Figure 3:**
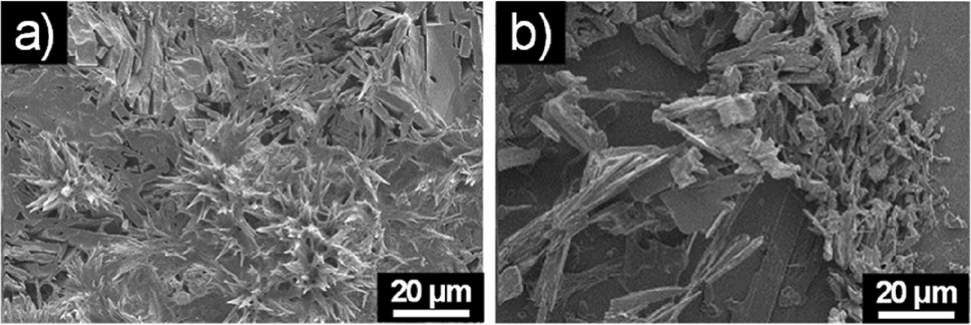
SEM micrographs of the self-assembled structures formed at basic pH by (**a**) **6a** and (**b**) **6b**.

The stability of **6a** and **6b** water aggregates was examined at different pH and upon heating at 120 °C. Both aggregates were not stable at high temperature and at acidic pH. At basic pH (pH = 10), a change of the morphology was observed (Fig. 3).

**Figure 4:**
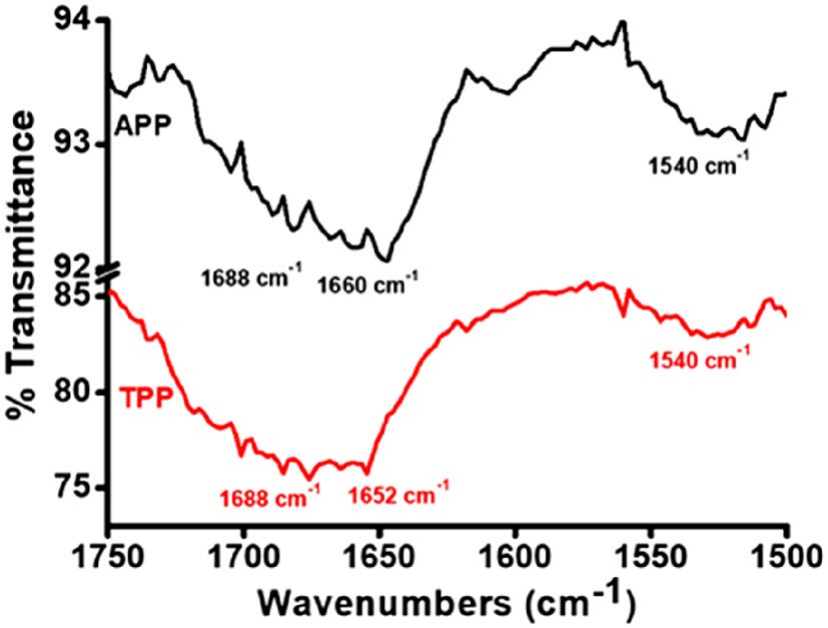
FT-IR spectra of the amide I and amide II regions of **6a** (red) and **6b** (black).

To understand the molecular conformation of the self-assembled structure of **6a** and **6b**, FT-IR experiments were performed (Fig. 4). In both cases, the minima at 1688 cm^−1^ in the amide-I region and 1540 cm^−1^ in the amide-II region suggested the presence of β-sheet conformations^33^. We thus hypothesized that the closer of sheets along the two axes results in the formation of the spherical structure^34^. The other minima in the spectrum (1652 cm^−1^ for **6a** and 1660 cm^−1^ for **6b**) are ascribable to adenine (A) and thymine (T) residues, respectively (see Fig. S2 in Supplementary Information).

**Figure 5:**
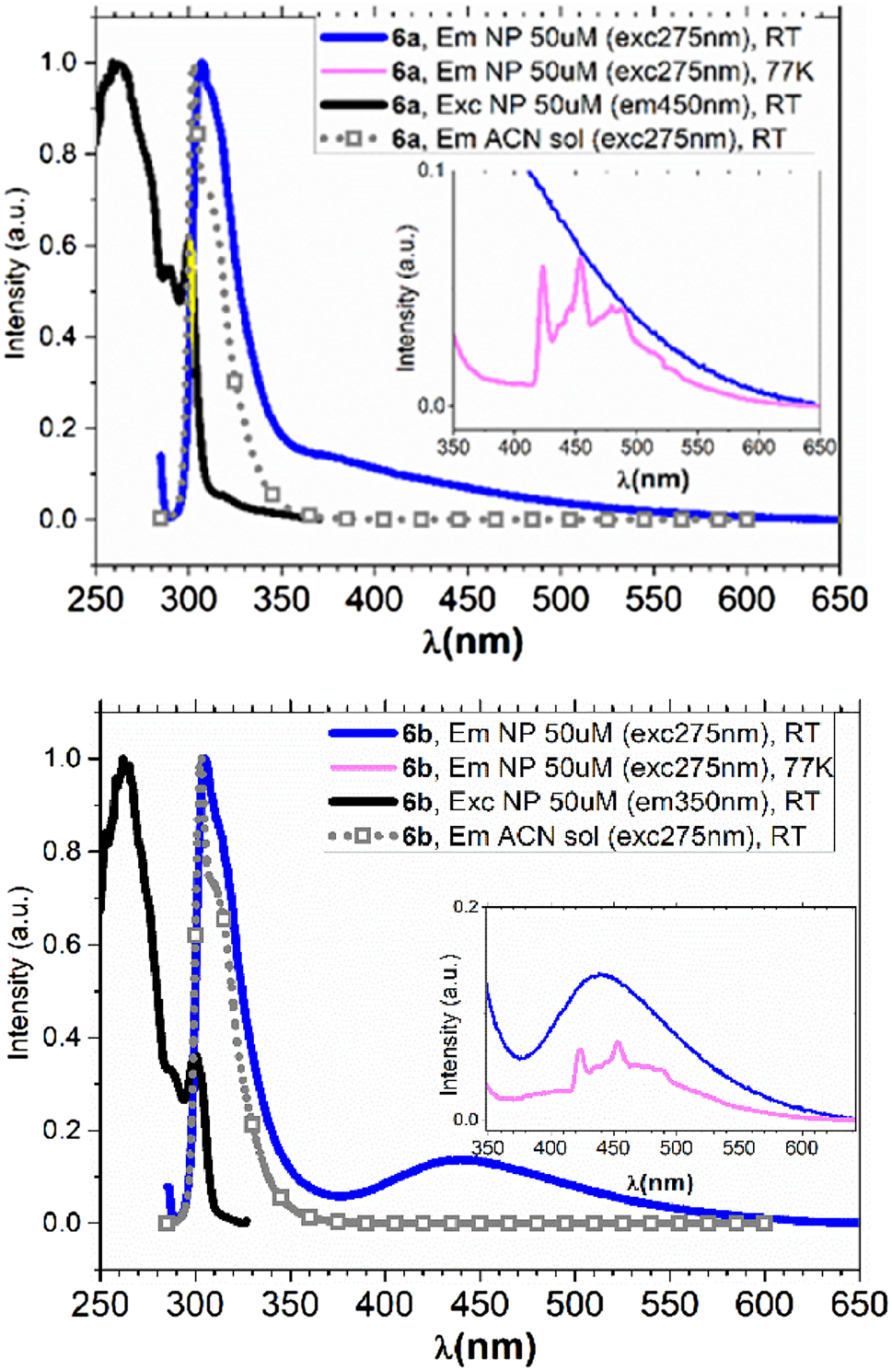
Emission and excitation spectra of **6a** (top panel) and **6b** (bottom panel) as diluted solution in ACN and as self-assembled NP at both RT and 77 K glass matrix.

Photophysical characterizations were performed on diluted solutions (1–2 × 10^–5^ M) of the tripeptides **6a** and **6b** (see Supplementary Information for details) and on self-assembled materials in water, obtained from HFIP-water (98:2 v/v) at the concentration of 5 × 10^−5^ M (Fig. 5). Photophysical characterization in the same conditions were performed on **1a** and **1b** (see Figs. S7, S8, Table S2 in Supplementary Information). The normalized absorption spectra of the tripeptides **6a** and **6b** acetonitrile (ACN) solutions (Fig. S3) looked very similar and are characterized by a strong deep UV absorption below 230 nm, a broad band between 240–280 nm and two slightly resolved vibrionic transitions between 280-300 nm (more evident in the excitation spectra, see Figs. S3 and S4 in Supplementary Information). In addition, both compounds **6a,b** showed deep blue room temperature photoluminescence in solution (PL, normalized data reported in Fig. 5 gray lines with square drawings and Fig. S4) with a maximum at 303 nm and a barely resolved shoulder at lower energy. The corresponding fluorescence quantum yield (QY) was 0.12 for **6a** and 0.13 for **6b**; the lifetimes were 5.0 and 5.5 ns respectively. The emissions and the photophysical parameters are similar to the ones measured on the Fmoc protected **1a** and **1b** (see Fig. S8 and Table S2). In Fig. 5, the PL emissions of the colloids obtained from HFIP-water mixture at 50 µM concentration are superimposed. Compound **6a** showed an overall QY of 0.018 and biexponential decay of 1.41(63%) and 6.06 (37%) ns at 330 nm. Its emission was composed by a dominant high energy band around 308 nm (similar to the emission in ACN solution and only slightly red-shifted) followed by a week visible emission tailing up to 500 nm. **6b**, with an overall QY of 0.03, displayed a high energy emission at 306 nm characterized by a biexponential decay of 1.08(57%) and 7.07(43%) ns at 330 nm, and a broad unstructured band between 400–600 nm with maximum at 440 nm and lifetime of 2.82(27%) and 13.1(73%) ns. Whereas the former is similar to the molecular emission in diluted solution, the latter could be ascribed to the fluorescence emission linked to the restricted intramolecular rotation of the backbone as previously reported in the literature for the Phe–Phe system^25^.

Upon cooling the colloidal suspensions of compounds **6a,b** at 77 K (pink curve in Fig. 5 insets) a new, previously undescribed, emission appeared in the visible range between 420 and 550 nm and characterized by structurally resolved features. These emissions had a long radiative lifetime of 4.2 and 4.1 s for **6a** and **6b**, respectively, thus indicating a process originated from a triplet state. To understand the origin of the phosphorescence emission, PL studies were performed on the isolated building blocks to generate compounds **6** (i.e. H_2_N- Phe–Phe -OMe **5** and the Fmoc protected **1**). As evident from Fig. S5 (SI) a structured emission above 400 nm is only observed from the nucleobase thus allowing the attribution of the phosphorescence observed in the tripeptide systems to the adenine or thymine unit^35^.

In conclusion, a new class of β amino acids containing a morpholino ring and a nucleobase has been developed starting from ribonucleosides. Their synthesis takes advantage from a “one-pot” oxidative ribose ring-opening and reductive amination, followed by the oxidation of the primary alcohol of the sugar. The so obtained β amino acids have been used for the functionalization of Phe–Phe dipeptide leading to sub-micrometric aggregates possessing photoluminescent features of both fluorescence and phosphorescence type. Thus, it was proved that the here presented tripeptides possess the photoluminescent properties given by the β-AA and the self-assembly behaviour due to the presence of Phe–Phe. They hence represent promising tools for the development of bioinspired functional materials with applications not only in the biotechnology field but also in non-biological optoelectronic ones.

## Supplementary information

Supplementary Information
