# Supplementary material for: Nucleobase morpholino β amino acids as molecular chimeras for the preparation of photoluminescent materials from ribonucleosides
Source: Sci Rep. 2020 Nov 9;10:19331. doi: 10.1038/s41598-020-76297-7 (PMC7652887; doi:10.1038/s41598-020-76297-7)
Supplement: Supplementary file 1 — Supplementary Information [file 41598_2020_76297_MOESM1_ESM.docx]

**Nucleobase morpholino β amino acids as molecular chimeras for the preparation of photoluminescent materials from ribonucleosides.**

Raffaella Bucci^a^, Alberto Bossi^b,c^, Emanuela Erba^a^, Francesco Vaghi^a^, Abhijit Saha^d^, Sivan Yuran^d^, Daniela Maggioni^c,e^, Maria Luisa Gelmi^a^, Meital Reches^d^, Sara Pellegrino^a^*

^a^ DISFARM-Dipartimento di Scienze Farmaceutiche, Sezione Chimica Generale e Organica ‘‘A. Marchesini’’, Università degli Studi di Milano, Via Venezian 21, 20133, Milano, Italy

^b^ Istituto di Scienze e Tecnologie Chimiche “G. Natta” del Consiglio Nazionale delle Ricerche (CNR-SCITEC), via Fantoli 16/15, I-20138, Milano, Italy and SmartMatLab Center, via C. Golgi 19, I-20133, Milano.

^c^ SmartMatLab Center, via C. Golgi 19, I-20133, Milano.

^d^ Institute of Chemistry and the Center for Nanoscience and Nanotechnology, The Hebrew University of Jerusalem, Jerusalem, Israel

^e^ Dipartimento di Chimica, Università degli Studi di Milano, Via Golgi 19, 20133, Milano, Italy

**1. Table S1. Oxidant tested on compounds 4a,b S2**

**2. Experimental procedures for the synthesis of compounds 1-6 a,b S3-S8**

**3. Self-assembly studies S9**

**4. DLS analysis S10**

**5. Photoluminescence (PL) studies S13**

**6. Copy of NMR spectra of compounds 1-6 a,b S17-S24**

**Table S1. Oxidants tested on compounds 4a,b**

| **Entry** | **Compound** | **Oxidants** | **Yield** |
| --- | --- | --- | --- |
| **1** | **4a** | 10% NaOCl, TEMPO, KBr, TBABr | Traces |
| **2** | **4a** | BIAB, TEMPO | 90% |
| **3** | **4a** | CrO_3_ | Not reproducible |
| **4** | **4a** | RuO_4_ | - |
| **5** | **4a** | KMnO_4_ | 20% |
| **6** | **4b** | BIAB, TEMPO | 15%^a^ |
| **7** | **4b** | KMnO_4_ | - |
| **8** | **4b** | CrO_3_ | 70% |

^a^ Removal of Fmoc protecting group under prolonged reaction conditions

**Experimental procedures for the synthesis of compounds 1-6 a,b**

**General information**

Chemicals were purchased from Sigma Aldrich and were used without further purification. HPLC analysis were performed on Jasco PU-980 pump equipped with a UV–vis detector Jasco UV-975 (wavelength: 220 nm) and on a Kromasil 5-AmyCoat column (4.6 mm i.d. × 250 mm, 5 µm, AkzoNobel). Mass spectra were recorded on an LCQESI MS were recorded on a LCQ Advantage spectrometer from Thermo Finningan and a LCQ Fleet spectrometer from Thermo Scientific. The NMR spectroscopic experiments were carried out either on Varian MERCURY 300 MHz (300 and 75 MHz for ^1^H and ^13^C, respectively), or Bruker Avance I 500 MHz spectrometers (500 and 125 MHz for ^1^H and ^13^C, respectively). Chemical shifts δ are given in ppm relative to the CHCl_3_ internal standard, and the coupling constants *J* are reported in Hertz (Hz).

**Synthesis of compounds 3a,b**

**3a**

In a round bottom flask, thymidine (1 g, 4.1 mmol) was dissolved in MeOH (70 mL). NaIO_4_ (0.9 g, 4.14 mmol) and (NH_4_)_2_B_4_O_7_ (0.9 g, 4.7 mmol) were added. After 2h the mixture was filtered and the white solid was washed abundantly with methanol (200 mL). The liquid phase was treated with NaCNBH_3_ (1.3 eq., 0.3 g, 5.3 mmol). After 20 min, others 0.4 eq. of NaCNBH_3_ (0.1g, 1.6 mmol) were added to the mixture that was left reacting for 15 min. Subsequently, a quenching with HCl was done, until the pH 3. Finally, the solution was concentrated under reduced pressure. Pure compound **3a** (yield: 60%, 0.68 g, 2.46 mmol) was obtained after crystallization with MeOH.

^1^H NMR (300 MHz, D_2_O) *δ* 7.54 (s, 1H), 5.93 (dd, *J* = 4.34, 2.29 Hz, 1H), 4.15-4.10 (m, 1H), 3.74-3.62 (m, 2H), 3.47-3.22 (m, 2H), 3.20-3.00 (m, 2H), 1.8 (s, 3H) ppm;

^13^C NMR (75 MHz, D_2_O) *δ* 166.2, 150.9, 136.7, 112.1, 77.3, 74.4, 61.1, 43.7, 42.5, 11.5 ppm;

MS (ESI): calcd for C_10_H_15_N_3_O_4_ 241.11, found [M + H]^+^ 242.20.

Elemental Analysis: calcd for C_10_H_16_ClN_3_O_4_ C, 43.25; H, 5.81; N, 15.13, found C, 43.15; H, 5.93; N, 15.03

**3b**

In a round bottom flask, adenosine (1 g, 3.7 mmol) was dissolved in MeOH (70 mL). NaIO_4_ (0.8 g, 3.74 mmol) and (NH_4_)_2_B_4_O_7_ (1.3 g, 4.3 mmol) were added. After 2h the mixture was filtered and the white solid was washed abundantly with methanol (200 mL). The liquid phase was treated with NaCNBH_3_ (1.3 eq., 0.3 g, 4.7 mmol). After 20 min, others 0.4 eq. of NaCNBH_3_ (0.1g, 1.5 mmol) were added to the mixture, that was left reacting for 15 min. Subsequently, a quenching with HCl was done, until the pH nd 3. Finally, the solution was concentrated under reduced pressure. Pure **3b** (yield: 70%, 0.74 g, 2.6 mmol) was obtained after crystallization with MeCN.

^1^H NMR (300 MHz, D_2_O) *δ* 8.49 (s, 1H), 8.40 (s, 1H), 6.30-6.26 (m, 1H), 4.33-4.28 (m, 1H), 3.81-3.65 (m, 4H), 3.51-3.46 (m, 2H) ppm;

^13^C NMR (75 MHz, D_2_O) *δ* 150.1, 148.0, 144.9, 143.9, 118.7, 77.7, 74.5, 61.1, 44.0, 42.7 ppm;

MS (ESI): calcd for C_10_H_14_N_6_O_2_ 250.13, found [M + Na]^+^ 274.43.

Elemental Analysis: calcd for C_10_H_15_ClN_6_O_2_ C, 41.89; H, 5.27; N, 29.31, found C, 41.71; H, 5.39; N, 29.25

**Synthesis of compounds 4a,b**

**4a**

Compound **3a** (0.5 g, 2 mmol) was dissolved in DMF (10 mL) and the solution was cooled to 0 ℃. Fmoc-Succinimide (0.7 g, 2 mmol) and DIEA (0.5 g, 4 mmol, 0.7 mL) were added and the solution was slowly wormed up to r.t. and kept under stirring for 3 h. The solvent was removed under reduced pressure and the crude product was purified by flash chromatography (DCM:MeOH = gradient 0-100%) affording **4a** as white solid (yield: 85%, 0.79 g, 1.7 mmol).

^1^H NMR (300 MHz, CDCl_3_) *δ* 8.86 (brs, 1H), 7.77-7.74 (m, 2H), 7.55-7.53 (m, 2H), 7.42-7.30 (m, 4H), 7.2 (brs, 1H), 5.65 (brs, 1H), 4.62-4.26 (m, 2H), 4.22-4.10 (m, 2H), 3.86-3.60 (m, 4H), 2.85-2.79 (brs, 2H), 1.93 (s, 3H) ppm;

^13^C NMR (75 MHz, CDCl_3_) *δ* 163.3, 154.8, 149.6, 143.7, 143.6, 141.3, 134.9, 127.8, 127.2, 125.0, 120.0, 111.4, 79.0, 77.2, 72.2, 67.7, 67.1, 62.8, 47.2, 46.3, 43.9, 14.2, 12.5 ppm;

MS (ESI): calcd for C_25_H_25_N_3_O_6_ 463.17, found [M + Na]^+^ 486.94.

Elemental Analysis: calcd for C_25_H_25_N_3_O_6_ C, 64.79; H, 5.44; N, 9.07; found C, 64.68; H, 5.57; N, 9.01.

**4b**

Compound **3b** (0.5 g, 2 mmol) was dissolved in DMF (10 mL) and the solution was cooled to 0 ℃. Fmoc-Succinimide (0.7 g, 2 mmol) and DIEA (0.5 g, 4 mmol, 0.7 mL) were added and the solution was slowly wormed up to r.t. and kept stirring for 3 h. The solvent was removed under reduced pressure and the crude product was purified by flash chromatography (DCM:MeOH = 10:1 with 1% AcOH) affording **4b** as white solid (yield: 55%, 0.52 g, 1.1 mmol).

^1^H NMR (300 MHz, DMSO) *δ* 8.35 (s, 1H), 8.22 (brs, 1H), 7.99-7.78 (m, 2H), 7.70-7.60 (m, 2H), 7.49-7.26 (m, 6H), 5.72 (d, *J* = 10.3, 1H), 4.97-4.92 (m, 1H), 4.57-4.28 (m, 3H), 4.25-3.86 (m, 2H), 3.80-3.57 (m, 2H), 3.55-3.42 (m, 2H) ppm;

^13^C NMR (75 MHz, DMSO) *δ* 157.2, 156.6, 154.8, 153.3, 150.0, 144.2, 141.2, 139.9, 139.2, 129.1, 127.6, 125.6, 120.5, 119.1, 110.2, 78.8, 76.9, 67.5, 61.9, 47.3, 46.4, 45.1 ppm;

MS (ESI): calcd for C_25_H_24_N_6_O_4_ 472.19, found [M + H]^+^ 473.42.

Elemental Analysis: calcd for C_25_H_24_N_6_O_4_ C, 63.55; H, 5.12; N, 17.79; found C, 63.12; H, 5.25; N, 17.70.

**Synthesis of compounds 1a,b.**

**1a**

Compound **4a** (0.3 g, 0.6 mmol) was dissolved in a mixture of MeCN/H_2_O (1:1; 10 mL) and the solution was cooled to 0 ℃. TEMPO (0.03 g, 0.18 mmol) and (Diacetoxyiodo)benzene (0.43 g, 1.32 mmol) were added and the solution was slowly warmed up to r.t.. After 5h the solvent was removed under reduced pressure and the crude was purified by flash chromatography (DCM:MeOH = gradient 0-100%) affording the pure **1a** as white solid (yield: 90%, 0.26 g, 0.54 mmol).

^1^H NMR (300 MHz, CDCl_3_) *δ* 9.66 (brs, 1H), 7.74-7.71 (m, 2H), 7.53-7.51 (m, 2H), 7.39-7.25 (m, 4H), 6.2 (brs, 1H), 5.80-5.60 (m, 1H), 4.46-4.31 (m, 2H), 4.23-4.11 (m, 4H), 3.00-2.86 (m, 2H), 1.88 (s, 3H) ppm;

^13^C NMR (75 MHz, CDCl_3_) *δ* 169.9, 164.1, 154.7, 149.8, 143.5, 143.4, 141.34, 141.3, 135.3, 127.8, 127.2, 124.93, 124.92, 120.1, 111.7, 110.0, 78.9, 74.2, 68.2, 60.5, 47.1, 44.2, 29.6, 14.16, 12.4 ppm;

MS (ESI): calcd for C_25_H_23_N_3_O_7_ 477.15, found [M + Na]^+^ 499.94; [M - H]^-^ 476.34.

Elemental Analysis: calcd for C_25_H_23_N_3_O_7_ C, 62.89; H, 4.86; N, 8.80; found C, 62.95; H, 4.93; N, 8.72.

**1b**

Compound **4b** (0.47g, 0.4 mmol) was placed in a round bottom flask with a stirrer bar. Under N_2_ flow, Acetone (5 mL) was added and the suspension was cooled to 0 ℃. Jones reagent (0.7 mL) was added and the reaction was warmed up to r.t. and kept stirring for 3h. The solvent was removed under reduced pressure and the crude product was purified by flash chromatography (DCM:MeOH = 10:1 with 1% AcOH) affording the product **1b** as white solid (yield: 70%, 0.14 g, 0.28 mmol).

^1^H NMR (300 MHz, DMSO) *δ* 13.20 (brs, 1H), 8.33 (s, 1H), 8.18 (brs, 1H), 7.88-7.80 (m, 2H), 7.68-7.58 (m, 2H), 7.39-7.20 (m, 4H), 5.79-5.76 (m, 1H), 4.60-4.0 (m, 4H),3.72-3.60 (m, 1H), 3.48-3.34 (m, 2H), 3.03-3.20 (m, 1H) ppm;

^13^C NMR (75 MHz, CDCl_3_) *δ* 169.5, 157.0, 155.0, 153.8, 150.0, 144.6, 141.6, 139.7, 128.5, 128.4, 128.0, 127.9, 125.9, 121.0, 119.4, 78.7, 76.7, 74.2, 68.1, 49.7, 47.5, 46.2, 45.0, 29.9, 29.5 ppm;

MS (ESI): calcd for C_25_H_22_N_6_O_5_ 486.17, found [M + Na]^+^ 511.12

Elemental Analysis: calcd for C_25_H_22_N_6_O_5_ C, 61.72; H, 4.56; N, 17.28; found C, 61.62; H, 4.60; N, 17.21.

**General condition for peptide coupling reaction**

The Morph-β AA **1a/1b** (1 eq.) was dissolved in DMF (0.1 M) and the solution was cooled to 0 ℃. HOBT (1.1 eq) and HBTU (1.1 eq.) were added and the solution was kept stirring for 1h. After that, NH_2_-PhePheCOOMe **5** (1 eq.) and DIEA (2.2 eq.) were added and the reaction was warmed up to r.t. and kept stirring overnight.

The solvent was removed under reduced pressure and the obtained yellow solid was dissolved in EtOAc and washed three times with water. The organic layer was dried over NaSO_4_ and concentrated under reduced pressure. The crude mixture was purified by chromatography column with *n*-Hexane:AcOEt (gradient 0-100%) affording the tripeptides **6** as white solids.

**6a**

yield: 82%; *m.p.*= 130 ℃..

^1^H NMR (500 MHz, CD_3_CN) *δ* 9.19 (brs, 1H), 7.85 (d, *J* = 5.35, 2H), 7.64 (d, *J* = 6.91, 2H), 7.48-7.12 (m, 14H), 7.04-6.60 (m, 2H), 5.77-5.72 (m, 1H), 4.75-4.55 (m, 2H), 4,55-4.40 (m, 2H), 4.36-4.25 (m, 1H), 4.20-3.90 (m, 2H), 3.70 (s overlapped, 3H), 3.67-3.59 (m overlapped, 1H), 3.23-3.08 (m, 3H), 3.07-2.25 (m, 3H), 2.64-2.46 (m, 1H), 1.96 (s, 3H) ppm;

^13^C NMR (125 MHz, CD_3_CN) *δ* 171.9, 170.6, 167.1, 163.7, 154.9, 150.3, 144.4, 141.6, 137.4, 137.0, 135.9, 129.9, 129.7, 128.8, 128.7, 128.1, 127.6, 127.2, 127.1, 125.5, 117.6, 111.0, 96.4, 78.9, 75.7, 67.7, 66.0, 55.5, 53.9, 53.7, 52.2, 47.5, 45.6, 44.7, 43.4, 37.4, 12.3 ppm:

MS (ESI): calcd for C_44_H_43_N_5_O_9_ 785.31, found [M + H]^+^ 786.34.

Elemental Analysis: calcd for C_44_H_43_N_5_O_9_ C, 67.25; H, 5.52; N, 8.91; found C, 67.15; H, 5.55; N, 8.83.

**6b**

yield: 75%; *m.p.*= 137 ℃.

^1^H NMR (500 MHz, CD_3_CN) *δ* 8.34 (brs, 1H), 8.10 (brs, 1H), 7.94 (d, *J* = 8.42, 1H), 7.84-7.77 (m, 1H), 7.72 (d, *J* = 8.42, 1H), 7.64 (d, *J* = 6.87, 1H), 7.56 (t, *J* = 6.87, 1H), 7.50-7.31 (m, 3H), 7.31-7.07 (m, 10 H), 7.04 (d, *J* = 8.42, 1H), 6.91 (d, *J* = 7.73, 1H), 6.48 (brs, 2H), 5.75 (brs, 1H), 4.71-4.48 (m, 3H), 4.36-4.25 (m, 1H), 4.19-3.97 (m, 1H), 3.70 (m, 1H), 3.67 (s, 3H), 3.55-3.42 (m, 1H), 3.22-2.75 (m, 4H) 2.66-2.53 (m, 1H) ppm;

^13^C NMR (125 MHz, CD_3_CN) *δ* 171.5, 170.1, 167.0, 155.1, 154,5, 151.60, 149.5, 146.4, 144.0, 141.1, 139.4, 137.0, 136.6, 129.4, 129.3, 129.2, 128.4, 128.3, 128.2, 127.7, 127.2, 127.1, 126.7, 126.6, 126.5, 125.0, 125.03, 124.4, 120.0, 119.1, 118.6, 109.7, 78.5, 74.8, 70.2, 67.3, 55.0, 53.56, 53.3, 51.8, 47.2, 46.0, 44.5, 37.0. ppm;

MS (ESI): calcd for C_44_H_42_N_8_O_7_ 794.32, found [M + Na]^+^ 817.12.

Elemental Analysis: calcd for C_44_H_42_N_8_O_7_ C, 66.49; H, 5.33; N, 14.10, found C, 66.41; H, 5.40; N, 14.00.

**Self-assembly studies**

**6a** or **6b** were dissolved in 1,1,1,3,3,3-Hexafluoro-2-propanol (HFP) at a concentration of 100 mg\mL. The peptide in HFP was diluted in different solvents (TDW, EtOH, 50% EtOH in TDW, MeOH, isopropanol, chloroform, TDW with NaOH pH=10, TDW with HCl pH=2) to a final concentration of 2 mg\mL: A drop from each sample was placed on a glass for SEM analysis at time=0 (right after dilution) and at time=24 (after overnight incubation).

After overnight incubation of APP\TPP in TDW, the temperature stability of the assemblies (the dried assemblies on a glass were heated to 120^◦^C for 2 hours) and the PH stability (the right amount of base\acid was added to the solution of the assemblies until 1M concentration of NaOH and HCl and the samples were left for an additional day of incubation) were studied by SEM analysis.

SEM images were taken using a Sirion high resolution scanning electron microscope or extra-high-resolution scanning electron microscope, MagellanTM400L, operating at 5kV.





Fig. S1. Spherical aggregates of **6a** in 50% EtOH in water.


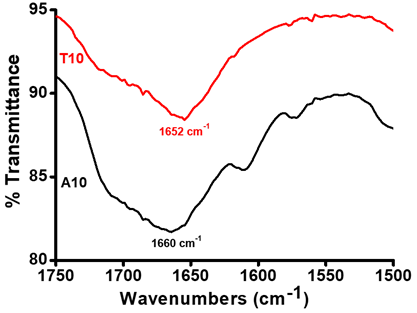


Fig. S2. FT-IR spectra of **Adenine** (black) and **thymine** (red).

**DLS analysis**

The Dynamic Light Scattering (DLS) measurements were performed using a Malvern Zetasizer Nano ZS instrument at 25° C, equipped with a 633 nm solid state He–Ne laser at a scattering angle of 173°. Analyses were performed in water (viscosity: 0.8872 Cp, refractive index: 1.33). The size measurements were averaged from at least three repeated measurements.

**6b** and **6a** were separately dissolved in hexafluoropropanol/H_2_O in 30:70, 20:80, 10:90 and 2:98 ratios respectively, such that the final concentration of 6a or 6b was equal to 50 mM for all the samples.

DLS showed some differences between **6a** and **6b**. In particular, at the lowest water content, the DLS size distribution by intensities for **6b** showed one peak centred at ca 5-6 nm attributable at few molecules forming small clusters, while in the case of **6a**, besides the peak at 5-6 nm, there was a second peak centred at 200-300 nm. Anyhow, this second population can be ascribed to a few aggregates already present, which accounts for a very minor part of the whole molecules present in the mixture, so that they can be neglected.

This condition was observed for **6b** only increasing the water in the mixture up to the 80%, while at the ratio 20:80 the **6a** already showed the bigger peak at ca 200 nm only, suggesting a more favourable aggregation capacity.


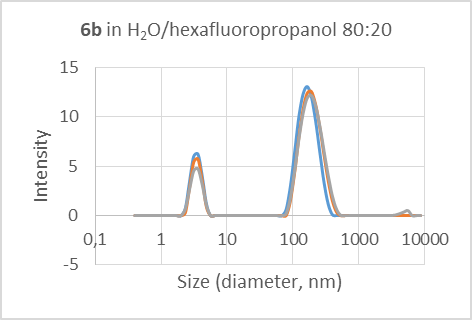

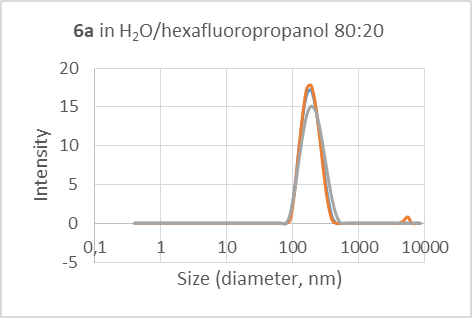


Both the two samples showed a huge increase in the size of the aggregates further increasing the water content at 10:90 ratio. Nevertheless, in this condition, the fitting of the correlation function was poor, so that the distribution output should not be considered reliable (data not shown).

When the content of hexafluoropropanol was decreased to 2% only the two suspensions showed 1 peak only centred at 200 nm for both **6a** and **6b**, suggesting that at time 0, the two nucleo-dipeptides behave at the same way. Moreover, compared to the peaks observed at 20:80 ratio, the peaks are sharper, indicating a more monodispersed situation.


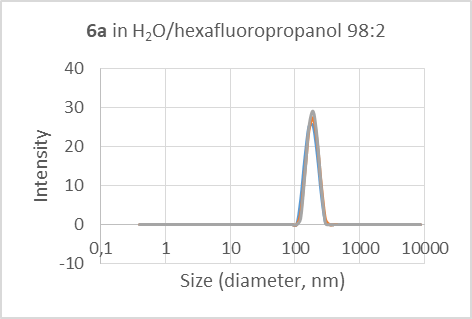

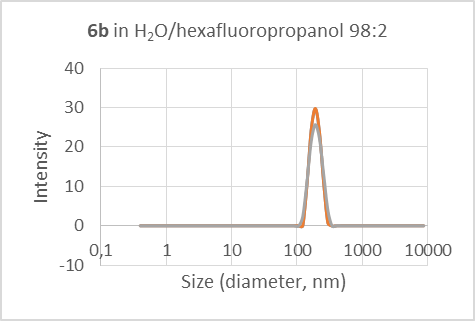


The stability of the aggregates over time was investigated acquiring a measurement at 24 h and 48 h.

| **H_2_O/hexafluoropropanol** | **6a** | **6b** |
| --- | --- | --- |
| **70:30** | 5.6 nm ± 2.0 (+ 260 ± 90 nm) | 5.6 ± 2.5 nm |
| **80:20** | 200 ± 98 nm | 3.6 ± 1.5 nm (+ 200 ± 85 nm) |
| **90:10** | --- | --- |
| **98:2 t=0** | 190 ± 60 nm | 190 ± 91 nm |
| **98:2 t=24h** | 342 ± 120 nm | 220 ± 100 nm |
| **98:2 t=48h** | 342 ± 150 nm | 220 ± 100 nm |
| **ζ-potential** | -47 mV | -16 mV |

**6a**

**6b**

**Photoluminescence (PL) studies**

**Absorption and emission measurements.** UV/Vis absorption spectra were obtained on Shimadzu UV-Vis-NIR 3600 Spectrophotometer in 1 cm path length quartz cell. Photoluminescence quantum yields were measured with a C11347 Quantaurus – QY Absolute Photoluminescence Quantum Yield Spectrometer (Hamamatsu Photonics U.K), equipped with a 150 W Xenon lamp, an integrating sphere and a multi-channel detector. Steady state emission and excitation spectra and photoluminescence lifetimes were obtained with a FLS 980 spectrofluorimeter (Edinburg Instrument Ltd.). Continuous excitation for the steady state measurements was provided by a 450 W Xenon arc lamp. Photoluminescence lifetime measurements were determined by TCSPC (time-correlated single-photon counting) method, were performed using an Edinburgh Pulsed Diode PLED-300 (Edinburg Instrument Ltd.), with central wavelength 300 nm and repetition rates 100ps or greater or for the long phosphorescence components by decay kinetics.

Photoluminescence experiments at room temperature were carried out in various solvent solution at 1-2x10^-5^ mol L^-1^ including acetonitrile (ACN), dimethylsulphoxyde (DMSO), water, methanol-ethanol (MeOH-EtOH) mixture. Measurements at 77 K were performed in MeOH-EtOH 4/1 mixture frozen matrix or by cooling the colloidal suspension obtained from HFIP-water at the concentration of 5x10^-5^ mol L^-1^.

**Fig. S3. Absorption spectra of compounds 6a and 6b in acetonitrile (ACN) solution.**

**Fig. S4. Emission, excitation and absorption spectrum of 6a (left) and 6b (right) in ACN solution at RT.**

**Fig. S5. Emission spectra of building blocks 5 and the 1b, 77K glassy matrix in MeOH/EtOH solution.**

**Fig. S6 Emission spectra of 6a and 6b in diluted ACN solution excitation and emission of the aggregated NP at RT and in frozen matrix t 77K.**

**Fig. S7. Absorption spectra of compounds 1a and 1b: left, molar absorptivities in acetonitirile (ACN) solutions; right, normalized ACN spectra (solid lines) compared to those obtained from a 98/2 v/v HFIP-water mixture at 50µM concentration (dashed lines with drawings).**

**Fig. S8: emission and excitation spectra of compounds 1a (left) and 1b (right): in both case are compared the curves collected from a diluted CAN solution and those obtained from a 98/2 v/v HFIP-water mixture at 50µM concentration.**

**Table S2: Photoluminescent emission properties in diluted ACN solutions and in 98/2 v/v HFIP-water mixture at 50µM concentration**

| *Compound (solvent)* | *QY* | *τ (%) / ns* |
| --- | --- | --- |
| **1a**, ACN solution | 0.137 | 4.34 |
| **1a**, 98/2 v/v HFIP-water mixture at 50µM concentration |  | 1.54(79%); 3.94(21%) |
| **1b**, ACN solution | 0.08 | 0.32(12%); 4.75(88%) |
| **1b**, 98/2 v/v HFIP-water mixture at 50µM concentration |  | 0.55(26%); 5.73(74%) |

**NMR Spectra**

**^1^H and ^13^C of compound 3a**


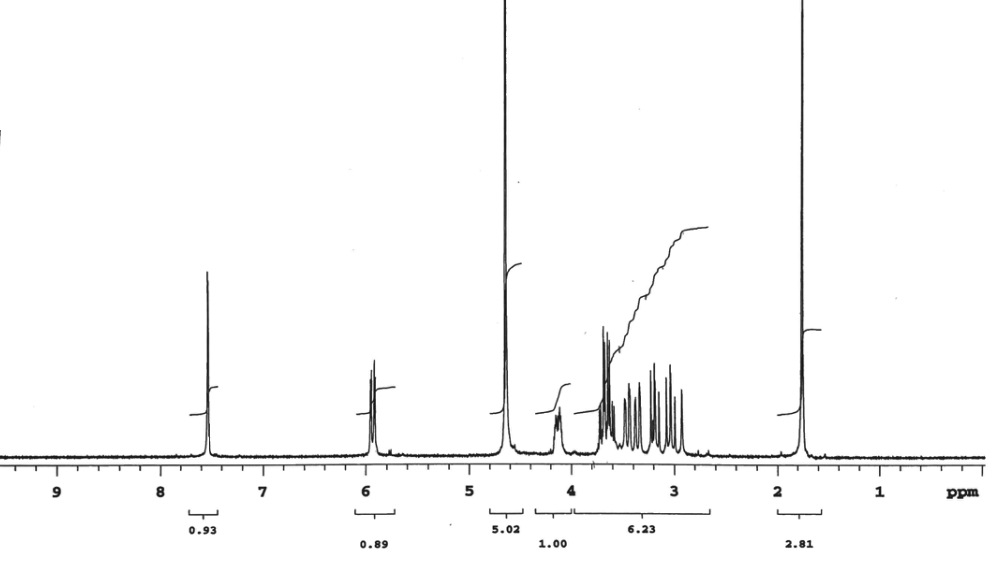


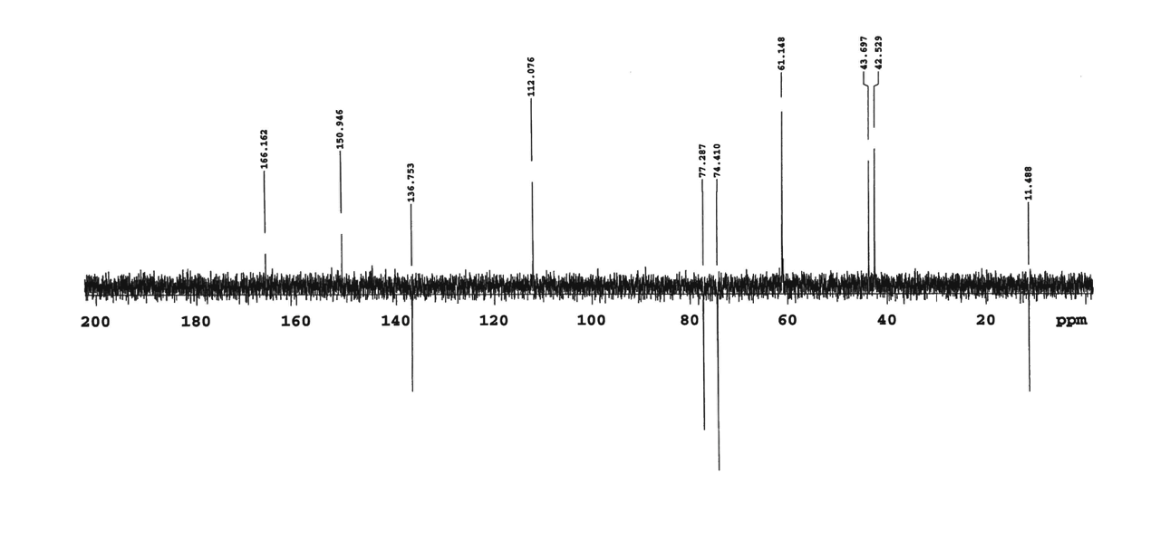


**^1^H and ^13^C of compound 3b**


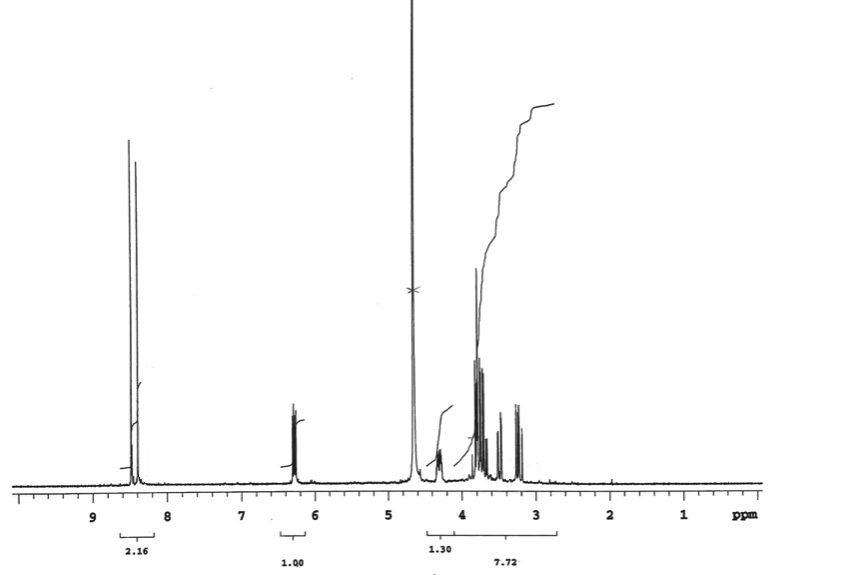


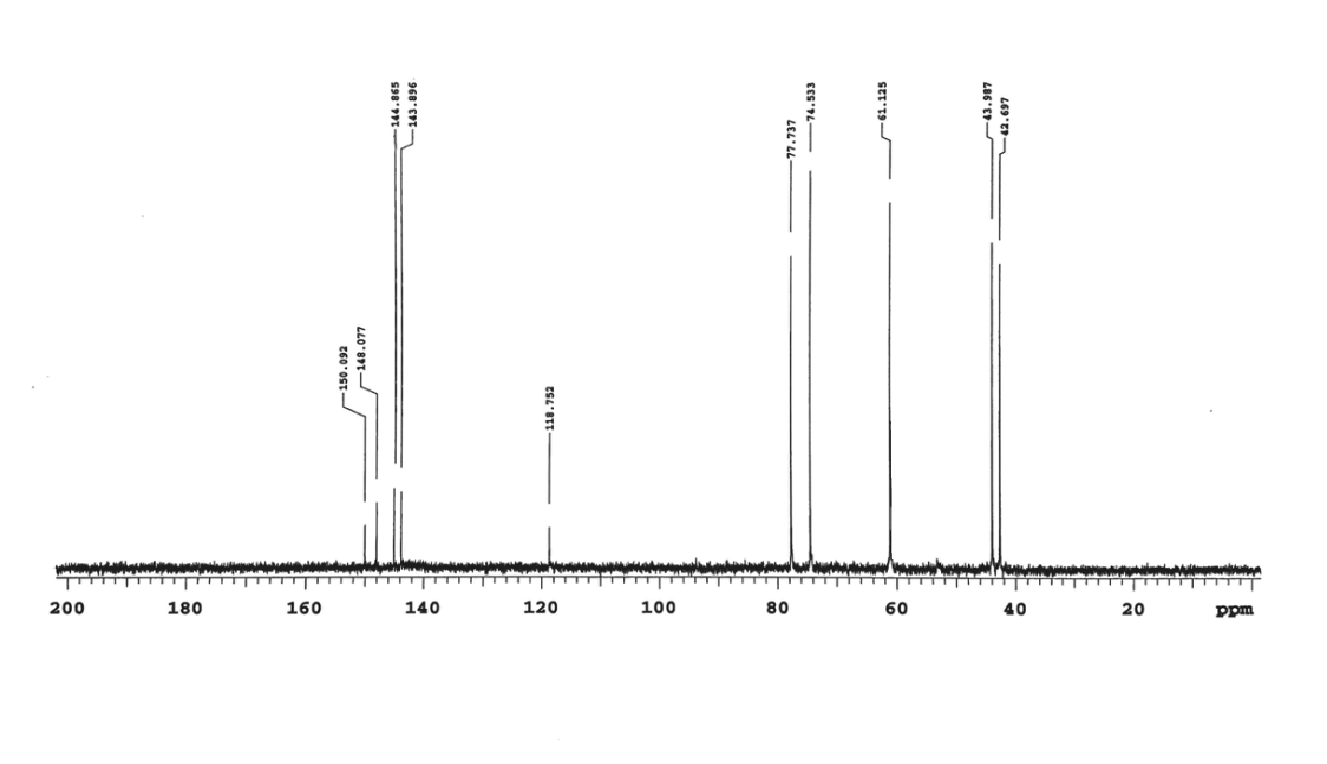


**^1^H and ^13^C of compound 4a**

**
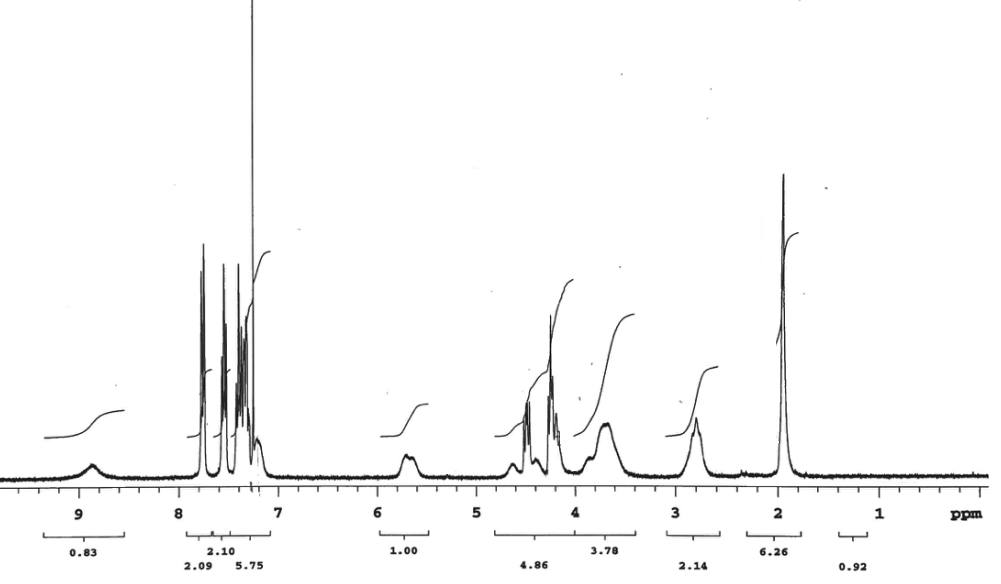
**


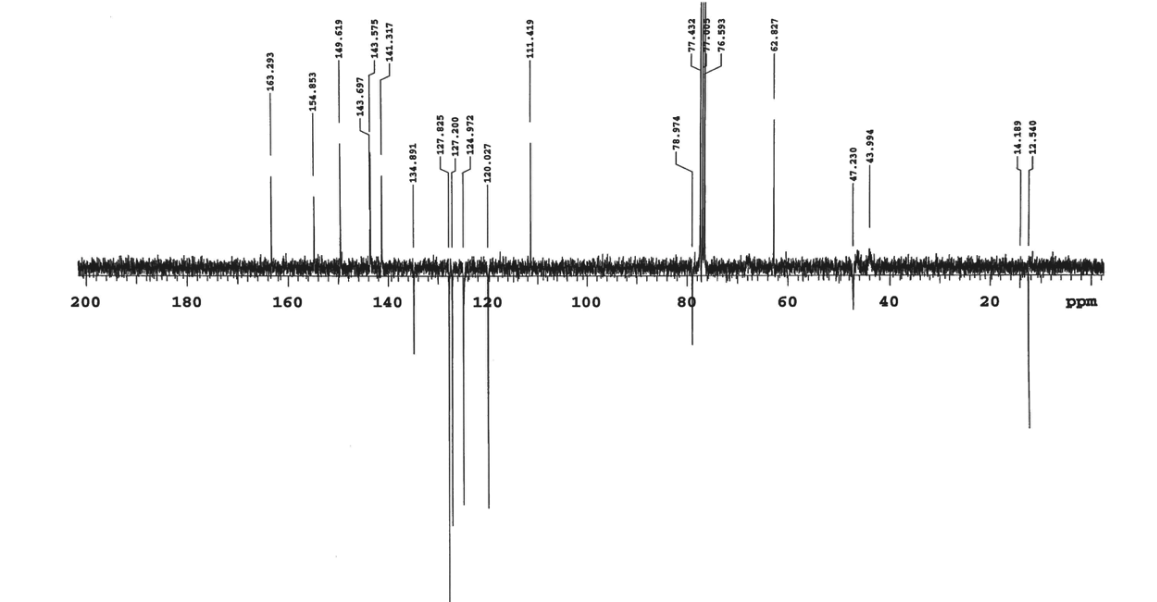


**^1^H and ^13^C of compound 4b**

**
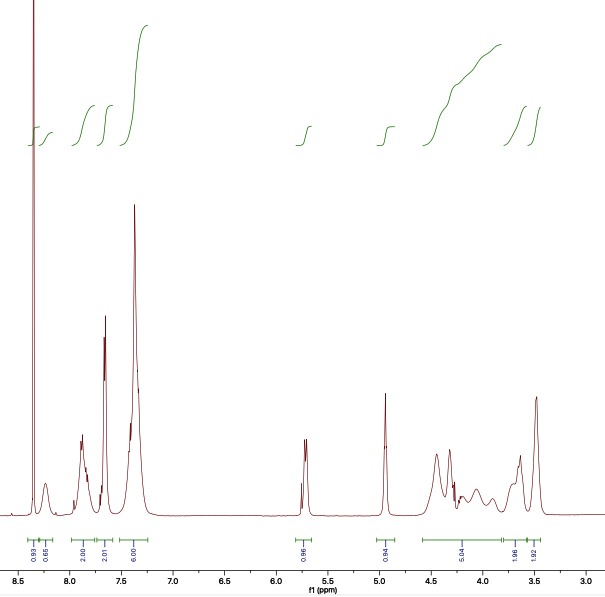
**


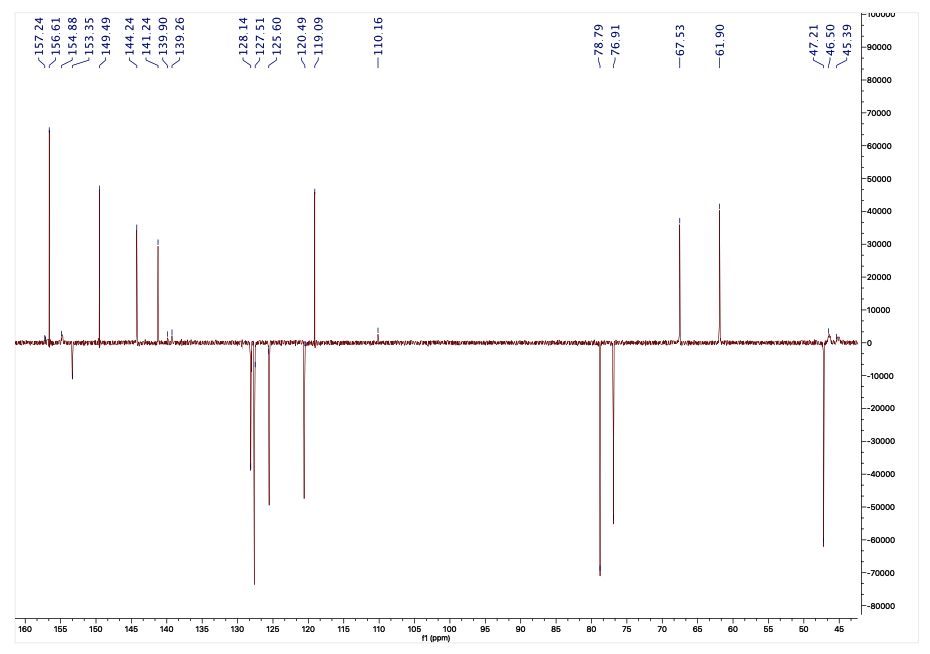


**^1^H and ^13^C of compound 1a**


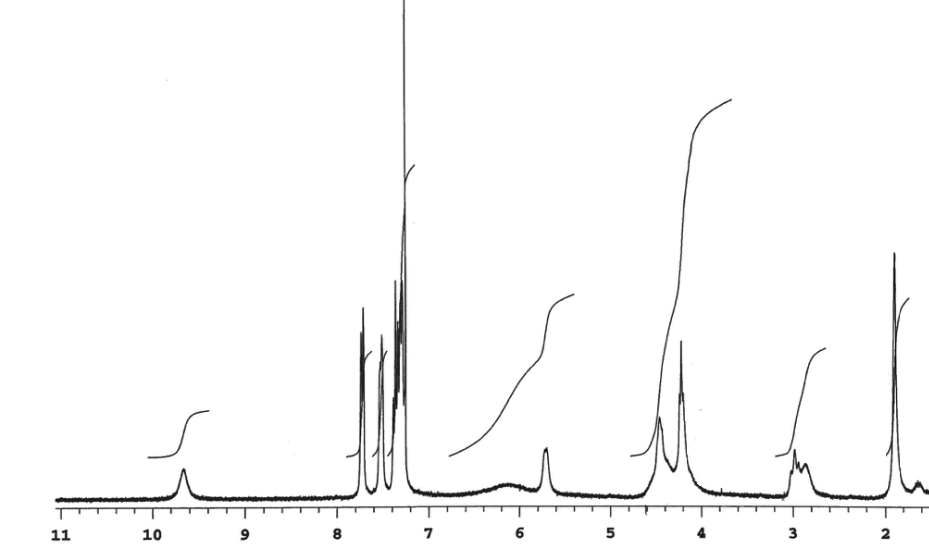


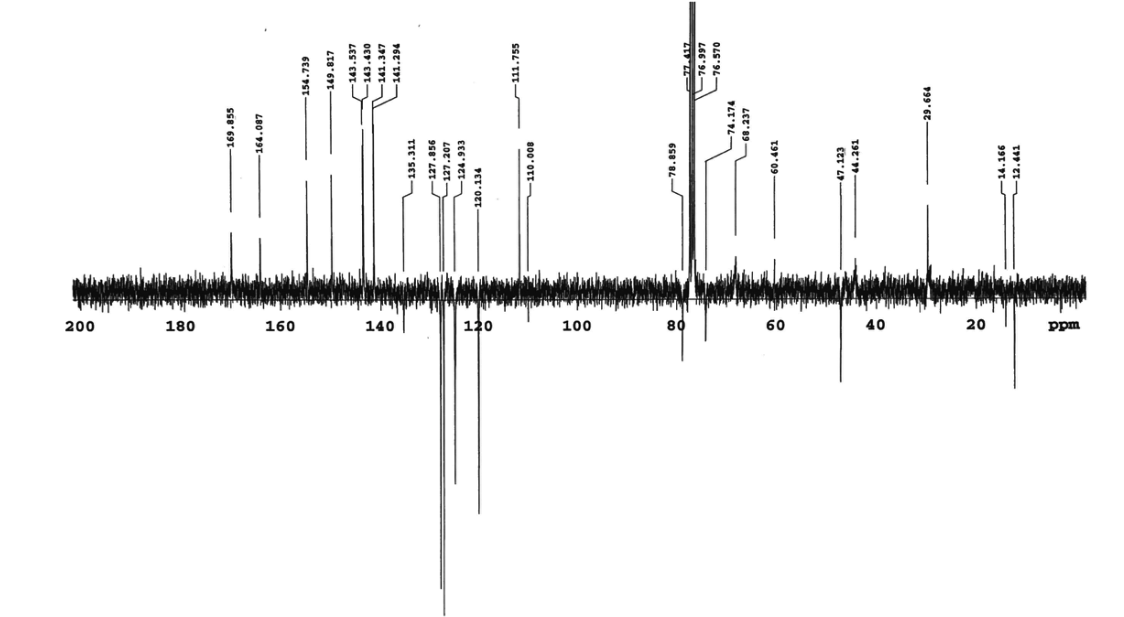


**^1^H and ^13^C of compound 1b**

**
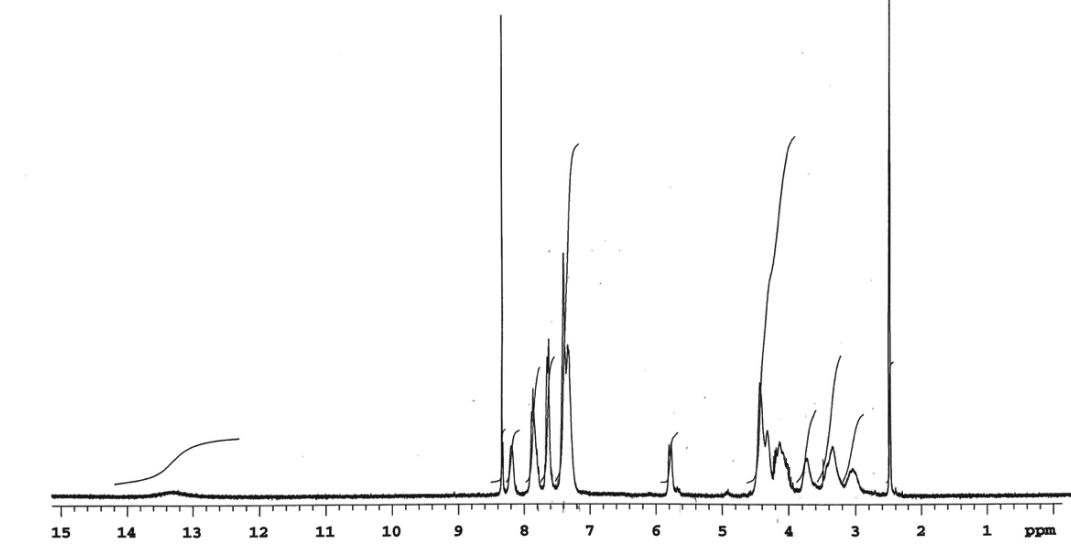
**

**
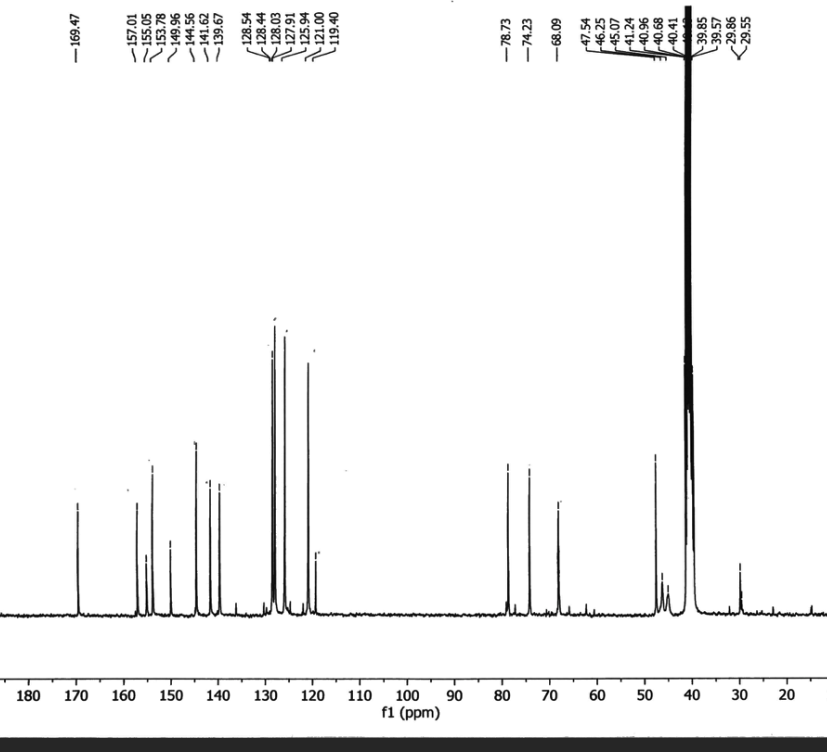
**

**^1^H and ^13^C of compound 6a**

**
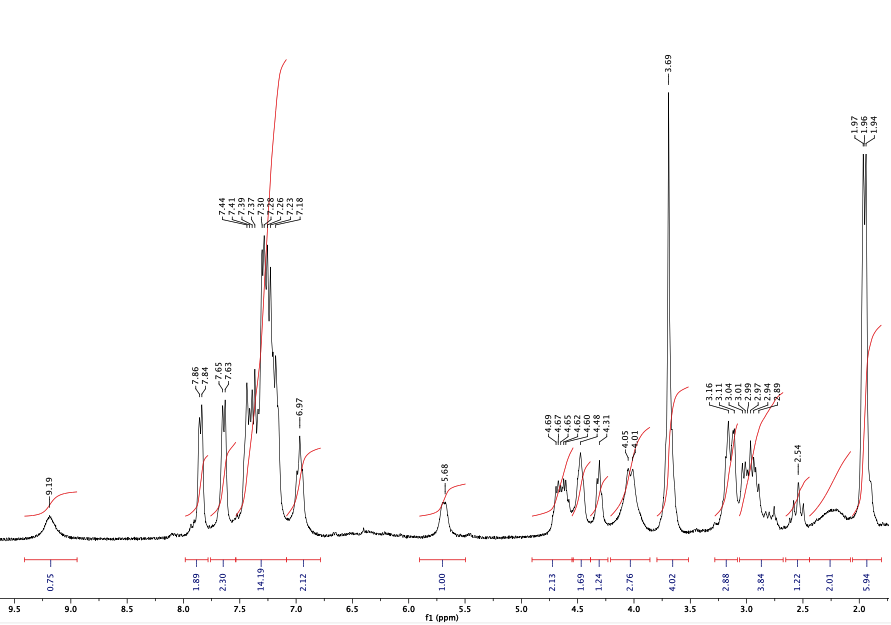
**

**
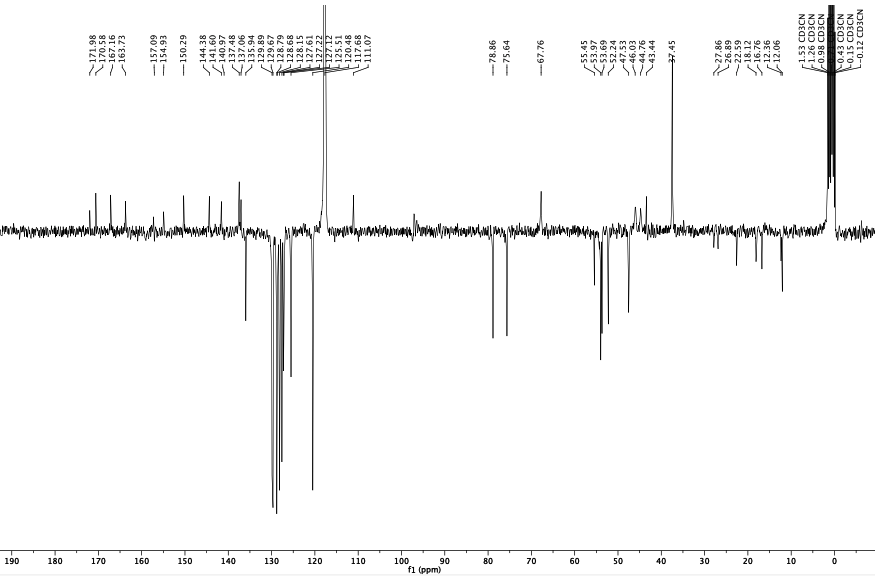
**

**^1^H and ^13^C of compound 6b**

**
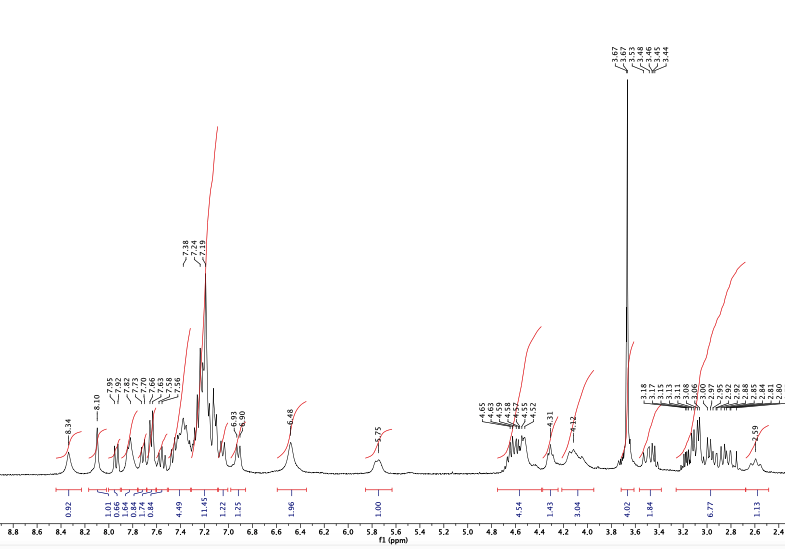
**

**
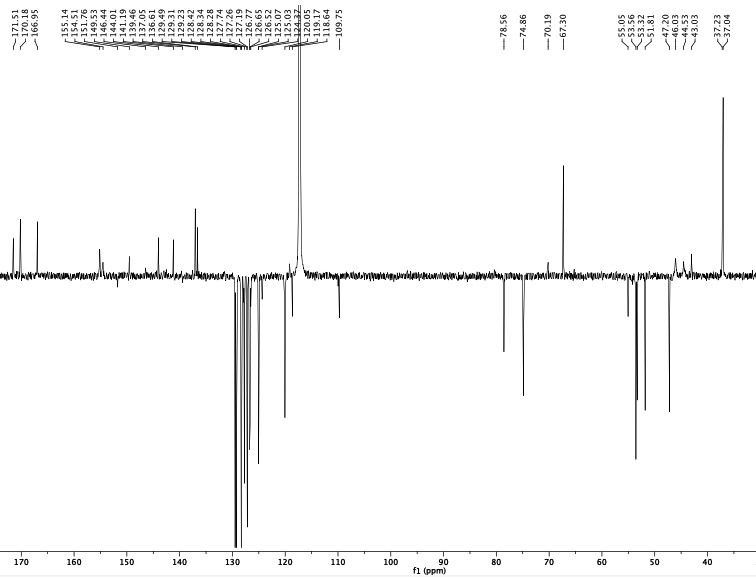
**
